# Staphylococcal Biofilms: Challenges and Novel Therapeutic Perspectives

**DOI:** 10.3390/antibiotics10020131

**Published:** 2021-01-29

**Authors:** Christian Kranjec, Danae Morales Angeles, Marita Torrissen Mårli, Lucía Fernández, Pilar García, Morten Kjos, Dzung B. Diep

**Affiliations:** 1 Faculty of Chemistry, Biotechnology and Food Science, The Norwegian University of Life Sciences, 1432 Ås, Norway; christian.kranjec@nmbu.no (C.K.); danae.morales.angeles@nmbu.no (D.M.A.); marita.torrissen.marli@nmbu.no (M.T.M.); 2 Department of Technology and Biotechnology of Dairy Products, Dairy Research Institute of Asturias (IPLA-CSIC), 33300 Villaviciosa, Spain; lucia.fernandez@ipla.csic.es (L.F.); pgarcia@ipla.csic.es (P.G.); 3 DairySafe Group, Instituto de Investigación Sanitaria del Principado de Asturias (ISPA), 33011 Oviedo, Spain

**Keywords:** *S. aureus*, coagulase-negative staphylococci, biofilm, bacteriocins, bacteriophages, antibiotics

## Abstract

Staphylococci, like *Staphylococcus aureus* and *S. epidermidis*, are common colonizers of the human microbiota. While being harmless in many cases, many virulence factors result in them being opportunistic pathogens and one of the major causes of hospital-acquired infections worldwide. One of these virulence factors is the ability to form biofilms—three-dimensional communities of microorganisms embedded in an extracellular polymeric matrix (EPS). The EPS is composed of polysaccharides, proteins and extracellular DNA, and is finely regulated in response to environmental conditions. This structured environment protects the embedded bacteria from the human immune system and decreases their susceptibility to antimicrobials, making infections caused by staphylococci particularly difficult to treat. With the rise of antibiotic-resistant staphylococci, together with difficulty in removing biofilms, there is a great need for new treatment strategies. The purpose of this review is to provide an overview of our current knowledge of the stages of biofilm development and what difficulties may arise when trying to eradicate staphylococcal biofilms. Furthermore, we look into promising targets and therapeutic methods, including bacteriocins and phage-derived antibiofilm approaches.

## 1. Introduction

Staphylococci are common inhabitants of the human skin microbiota, but they are also among the major pathogens responsible for hospital-acquired infections worldwide [1,2,3,4,5,6]. Their pathogenic potential stems from their ability to express a wide variety of virulence factors. Among these, there are genes involved in the formation of sessile bacterial communities known as biofilms. This form of bacterial life is characterized by three-dimensional communities of microorganisms that are surrounded by an extracellular matrix, also known as an extracellular polymeric substance (EPS). The EPS in staphylococcal biofilms consists of polysaccharides, extracellular DNA (eDNA) and/or proteins [7,8,9,10,11]. Additionally, the biofilm formation promotes adhesion onto a variety of biotic and abiotic surfaces, increasing the resilience of bacterial communities to hostile environmental factors (e.g., the presence of antibiotics or host immune responses). This is particularly dangerous in the case of pathogenic bacteria, as it makes their elimination even more challenging. An important characteristic of biofilms is that at later stages of maturation, they allow for dispersal of biofilm-associated bacterial cells, resulting in colonization of additional niches [12,13,14,15,16].

It has been estimated that biofilm formation is involved in 65–80% of bacterial infections in humans. Both Gram-positive and -negative pathogens can form biofilms during infection, with staphylococcal, *E. coli* and *Pseudomonas aeruginosa* biofilms being the most studied [17]. Among staphylococci, *S. aureus* and *S. epidermidis* are the most common etiological agents of prosthetic medical device infections in the US and Europe [18,19,20]. Indwelling medical devices are also particularly exposed to bacterial biofilm formation due to their surfaces, making them excellent supports for bacterial adhesion [21]. Intense research efforts have therefore been devoted to developing strategies to prevent biofilm formation on such surgical devices, either by passively preventing bacterial colonization (e.g., surface engineering) or by actively inhibiting bacterial growth (e.g., antimicrobial coating) [22,23,24,25,26,27]. In human infections, the main source of staphylococcal contamination is their natural presence on the human skin. It has, for instance, been estimated that *S. aureus* colonizes the vestibulum nasi (anterior nares) of 20 to 80% of the human population [28]. Consequently, staphylococci significantly contribute to a complex pattern of infections in humans, which includes, but is not limited to, surgical site and skin and soft tissue infections (SSTIs), bone and joint infections, bacteremia and endocarditis [29,30,31,32].

In clinically relevant situations, biofilm-associated infections often have a polymicrobial nature. Mixed biofilms substantially increase the complexity of the infection, by altering the antimicrobial susceptibilities of the species involved and making conventional treatments inadequate [33]. The formation of polymicrobial biofilms involving staphylococci can be favored by their interaction with several other microorganisms, including bacteria (Gram-positive and -negative), yeasts (i.e., *Candida albicans*) and viruses [34,35,36,37,38]. In addition, staphylococcal biofilm-associated infections with a polymicrobial nature involve several body districts; most notably, these include cystic fibrosis lung and diabetic foot infections [39,40].

The next sections will cover more in detail the staphylococcal virulence factors important for the formation of biofilms, and how the interaction between biofilm formation and antibiotic resistance causes particularly insidious infections. Finally, we will also review recent strategies and alternatives to conventional antibiotic therapies to treat such infections.

## 2. Development of Staphylococcal Biofilms

**Figure 1:**
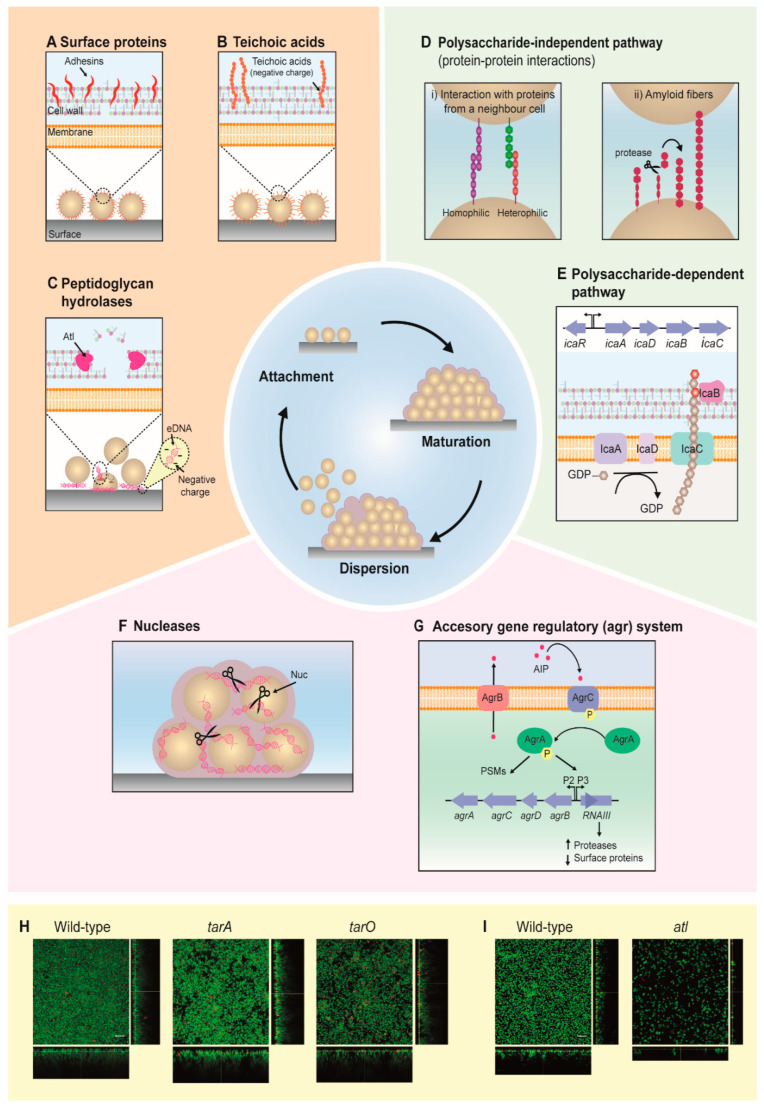
Stages and pathways involved in staphylococcal biofilm development. (**A**–**C**) During the attachment stage, an initial population of staphylococcal cells adhere onto a surface via (**A**) surface proteins, (**B**) teichoic acids and/or (**C**) eDNA released from cells, the latter due to cell lysis mediated by peptidoglycan hydrolases like AtlA/E and Sle1.(**D**–**E**) The maturation stage is mediated by intercellular adhesion, which allows the formation of multiple cell layers and the increase of the biofilm cell population. Intercellular adhesion is made via (**D**) a polysaccharide-dependent or (**E**) polysaccharide-independent pathway. The former involves homophilic or heterophilic protein-protein interactions by surface proteins (i) or amyloid fibers (ii). Certain surface proteins are processed by proteases to remove part of it, allowing the protein to polymerise into a fiber to reach a neighboring cell. The polysaccharide-dependent pathway is mediated by the *icaADBC* locus, which is responsible for the synthesis of β-1,6-N-acetylglucosaminoglycan (PIA/PNAG - grey hexagons). Then, PNAG chains are translocated to the outside of the cells, probably by IcaC, and deacetylated (red hexagons) by IcaB. (**F**,**G**) During the dispersion stage, cells are released from the biofilm. It has been suggested that the nuclease Nuc (**F**) participates in the degradation of eDNA during this stage. In addition, the accessory gene regulatory (*agr*) system (**G**) also plays a role during dispersion (please see the main text for details). (**H**,**I**) Examples of how the expression of selected genes affects *S. aureus* biofilms, imaged by confocal microscopy. Effects on *S. aureus* NCTC8325–4 biofilm formation when the genes *tarA* and *tarO* were depleted by using CRISPR interference (**H**), and on *S. aureus* JE by *Δatl* mutation (**I**). Biofilms were stained using a LIVE/DEAD kit. Green fluorescence indicates live cells, while red fluorescence indicates dead cells. Scale bar: 10 µm.

Biofilm development is a complex process involving a large number of staphylococcal proteins that can be divided into three general stages: attachment, multiplication/maturation and detachment/dispersion (Figure 1). In this section, we will give an overview of the main mechanisms that occur during the development of staphylococcal biofilms.

### 2.1. Attachment

The following section will provide an overview on the staphylococcal surface proteins that contribute to the attachment phase. Biofilm formation is initiated by cells attaching to a surface, which may be biotic (e.g., epithelial tissues) or abiotic (e.g., medical devices). Staphylococci have developed an extensive set of surface proteins with a characteristic LPXTG motif at the C-terminus, allowing sortase A-mediated attachment to the cell wall [41]. Particularly, many microbial surface component recognizing adhesive matrix molecules (MSCRAMMs) have been reported to be involved in the initial stages of attachment (Figure 1A) and biofilm formation in pathogenic staphylococci; for instance, the proteins Bap, SasC, FnBPA and FnBPB are expressed in *S. aureus* [42,43,44,45], and the proteins Aap, SSP1 and SSP2 in *S. epidermidis* [46,47]. Although these proteins bind different types of surfaces, they are structurally similar and share the same binding mechanism [48]. Several studies have shown that teichoic acids also play a key role in staphylococcal biofilm formation [49,50,51] (Figure 1B,H). Teichoic acids are glycopolymers anchored to the cell wall (CW) via covalent bonds to the CW components (wall teichoic acids) or alternatively to the cell membrane via lipid anchors (lipoteichoic acids). Notably, teichoic acids play an important role in bacterial colonization of abiotic surfaces, such as polystyrene and glass, where their net negative charge mediates surface adhesion [49].

Hydrolases, such as AtlA/E (AtlA and AtlE in *S. aureus* and *S. epidermidis*, respectively) and Sle1 also participate in the initial stage of attachment (Figure 1C,I) [52,53,54,55,56,57]. AtlA/E is a bifunctional murein hydrolase with amidase and glucosaminidase activities that also plays an essential role during cell division [58]; while Sle1 is suggested to be involved in the same process [59,60]. Two possible explanations as to how hydrolases such as AtlA/E are involved during attachment have been suggested. First, AtlA/E can act as an adhesin [53,54,61]; second, AtlA/E can degrade the cell wall, allowing the release of DNA, which in turn contributes to the formation of the adhesive EPS [62,63]. Furthermore, staphylococci encode a range of putative cell-wall hydrolases, in addition to AtlA/E and Sle1, which potentially can contribute to biofilm formation. Future research should aim at investigating the role of these hydrolases in biofilm development.

### 2.2. Maturation

After the initial attachment, a process known as maturation consolidates the transition from planktonic to sessile bacterial life. During this stage, the major part of the EPS is formed, promoting intercellular adhesion and allowing cells to accumulate in multiple layers to form a three-dimensional community. In staphylococci, the formation of the EPS can be achieved through two main pathways: a polysaccharide-dependent (Figure 1E) and a polysaccharide-independent pathway (Figure 1D).

The first molecule identified responsible for intercellular adhesion in the polysaccharide-dependent pathway was the extracellular polysaccharide intercellular adhesin (PIA) β-1,6-N-acetylglucosaminoglycan (PNAG) [64]. The synthesis of PIA/PNAG, the main component of the polysaccharide-dependent EPS in staphylococci, is performed by the four-gene locus *icaADBC* (intercellular adhesion), which is under transcriptional control by IcaR. This locus was first identified in *S. epidermidis* [54], and was later confirmed to be present in other *Staphylococcus* species as well [65]. The genes *icaA* and *icaD* encode membrane proteins that are responsible for the synthesis of the polysaccharide PIA/PNAG [66], which is exported by IcaC and subsequently deacetylated by the *icaB* gene product, a process important for the retention of polysaccharide onto the bacterial cell surface [11,66,67,68]. Furthermore, it has been suggested that IcaC is an O-succinyltransferase, an enzyme that might be involved in the succinylation of PNAG, a modification of the polysaccharide which is important for its functionality in vivo [69].

The polysaccharide-independent pathways often rely on protein-mediated intercellular adhesion. The accumulation-associated protein (Aap) in *S. epidermidis* was the first PIA-independent pathway reported [54]. Since then, many other pathway mediators have been identified, including *S. aureus* surface protein G (SasG) [70], biofilm-associated protein (Bap) [42] and fibronectin-binding protein A (FnBPA) [71]. Two different mechanisms of protein-protein interaction have been described. In the first one, proteins can interact via homophilic (same type of proteins) or heterophilic (different types of proteins) interactions with the proteins from a neighboring cell. Examples of proteins using this mechanism are Aap, SasG and FnBPA. These surface-exposed proteins are involved in Zn^2+^-dependent protein-protein interactions. Notably, Aap and SasG-mediated interactions are under proteolytic control, in which the exposure of the adhesion domain is dependent on a proteolytic cleavage mediated by proteases such as SepA [72], indicating that such mode of cell-cell interaction is highly regulated. In the second mechanism, proteins polymerize to form amyloid fibers that allow the bacteria to get in contact with the neighboring cells. An important mediator of this adhesion process is Bap, which assembles into amyloid fibers in a proteolytic cleavage-mediated process [73].

In addition to polysaccharide PIA and protein-protein interactions, the EPS in staphylococcal biofilms can also consist of extracellular DNA (eDNA), which with its negative electric charge potentially acts as an electrostatic polymer that anchors cells to surfaces, host factors and other cells. In these cases, this relies on the action of secreted hydrolases such as AtlA/E and Sle1 (see Section 2.1) to cause cell lysis and release of their DNA content.

Recent studies have also suggested the presence of an additional stage in the biofilm cycle, known as exodus. The exodus is characterized by the migration of a subpopulation of biofilm-associated cells soon after sessile bacterial cells begin to divide and accumulate [52]. The exodus stage is regulated by a secreted staphylococcal nuclease (encoded by *nuc*) [52], which is under the control of the Sae regulatory system [74]. The secreted staphylococcal nuclease digests eDNA, and as a consequence, some cells are released from the biofilm. Nuc expression seems to be regulated in a stochastic manner, as the *nuc* promoter is activated only in a small part of the population [52,75]. The exact role of exodus during biofilm development remains to be fully elucidated, including its role in biofilm (re)structuring [76], as well as the fate of the released staphylococcal cells.

### 2.3. Dispersion

Detachment or dispersion is the last stage of the biofilm formation cycle. During this stage, some of the components of the EPS are degraded to allow bacteria to be released from the biofilm. The detachment stage has a key role during dissemination of staphylococcal infections [77,78]. The dispersion of the biofilm matrix can be mediated by proteases [79,80,81] and nucleases, but also by proteins with surfactant activity [78].

It has been suggested that nucleases may be especially important during biofilm detachment when eDNA is one of the main components of the biofilm matrix. *S. aureus* has two secreted nucleases known as Nuc and Nuc2; expression of either one results in the inhibition of biofilm formation [82,83,84] (Figure 1F). However, a clear role of these proteins in dispersion has not been reported.

Proteases such as SplABCDEF [79], SspA (V8) serine protease [80] and aureolysin (Aur) [79,81] have been associated with the detachment of protein-dependent biofilms. The expression of these extracellular proteases is regulated by the accessory gene regulator (*agr*) system [79] (Figure 1G), which plays a central role in staphylococcal biofilm formation and pathogenesis. The *agr* system has been shown to control the expression of the phenol-soluble modulins (PSMs)—a group of peptides with surfactant properties that are involved in the dispersion stage [78]. In general, Agr is a peptide-based quorum-sensing system that senses the density of the cells through the extracellular autoinducing peptide (AIP) [85]. During their growth, staphylococcal cells produce and secrete AIP, leading to its accumulation in the extracellular environment. When the extracellular AIP concentration reaches a critical threshold, it binds to the membrane-bound histidine kinase AgrC, triggering its autophosphorylation [86]. This activates a phosphoryl group transfer from AgrC to its cytosolic partner, the transcription factor AgrA, which, in turn, activates gene expression [87]. One of the transcriptional targets regulated by AgrA is RNAIII, a small RNA molecule that regulates the expression of hundreds of downstream genes, including biofilm-associated proteases and other virulence factor genes [88,89]. In this context, recent studies indicated that a *C. albicans*-mediated enhanced induction of *agr* in *S. aureus* correlated with increased mortality rates in coinfection patients [90].

### 2.4. Biofilm Formation Is Highly Dependent on Environmental Factors

The plethora of proteins involved in the process of biofilm formation underlines the complexity of this process. The nature of the EPS and the pathways involved rely on environmental factors such as the nature of the surface on which the biofilm is formed, the nutrients and electron acceptors available and other factors present in the local microenvironment. Biofilm-associated cells are thus able to sense the environmental cues to communicate and coordinate biofilm formation with neighboring bacterial cells. Such communication is often controlled by quorum-sensing (QS) mechanisms, which coordinate and fine-tune gene expression during the biofilm cycle [91,92,93,94,95,96]. The Agr-system described above is just one of them. Other regulatory systems, such as the two components systems SrrAB and SaeRS have been shown to sense the respiratory status of the bacterial cells and, under oxygen-limiting conditions, induce expression of AtlA, resulting in increased cell lysis and formation of fermentative biofilms [63,97]. For a more detailed description of regulatory pathways involved in staphylococcal biofilms, readers are referred to a recent review by Schilcher and Horswill [98], who have excellently treated this topic.

## 3. Hurdles in Tackling Staphylococcal Biofilms

Like other staphylococcal infections, biofilm-associated infections are often treated with conventional antibiotics. Upon diagnosis of staphylococcal infections, however, therapeutic options are often limited due to the widespread resistance mechanisms developed by these bacteria (see below). The high propensity of staphylococci to form biofilms confers additional competitive advantages, which include a 10- to 1000-fold increase in resistance to antibiotics compared to planktonic cells [99], further elevating their resilience to treatment. In this section, we will discuss different problems encountered when treating staphylococcal biofilms with antibiotics, including the antibiotic resistance spread, the limited penetration of antibiotics into biofilms, the heterogeneity of cells in a biofilm community and the potential for horizontal transfer of antibiotic resistance genes within biofilms.

### 3.1. Antibiotic Resistance among Pathogenic Staphylococci

Ever since the introduction of antibiotics in human medicine, staphylococci have been infamously known for their ability to develop resistance against these antimicrobial molecules. In the 1950s, only a few years after the introduction of penicillin, a β-lactam antibiotic acting on the penicillin-binding proteins (PBPs) of the *S. aureus* cell wall, β-lactamase-expressing staphylococcal strains began to be reported, soon conferring a widespread resistance to this β-lactam [100]. Furthermore, the introduction of methicillin, a synthetic β-lactamase-resistant penicillin, was soon followed by a rise in methicillin-resistant *S. aureus* (MRSA) strains. These strains had horizontally acquired an alternative penicillin-binding protein (PBP2B) with reduced affinity for β-lactams, thus making them resistant to most antibiotics of this class [101,102,103,104]. Similarly, the introduction of glycopeptide antibiotics, such as vancomycin, another group of cell-wall-targeting antibiotics, has resulted in the emergence of vancomycin-intermediate *S. aureus* (VISA) [105], and more recently of vancomycin-resistant *S. aureus* (VRSA) strains [106,107], which are tolerant or resistant, respectively, to vancomycin [101,108,109,110]. Importantly, extrinsic factors also play a role in the modulation of antibiotic susceptibility in staphylococci. In this regard, mixed biofilms with *C. albicans* have been shown to confer *S. epidermidis* and *S. aureus* cells a specific and enhanced tolerance toward vancomycin and other antimicrobials, linked to the protective effect provided by the fungal ECM and modulation of the drug metabolism in bacterial cells [38,111,112,113]. Similarly, in mixed biofilms, *S. aureus* and *P. aeruginosa* have been shown to bidirectionally alter their respective antimicrobial susceptibility patterns [114,115,116].

The intense use of antibiotics in the second half of the 20th century led to the selection of multi-drug-resistant (MDR) bacterial strains; such as MRSA strains showing resistance also to macrolides, aminoglycosides and/or fluoroquinolones [108]. The resistance mechanism in these cases can range from the expression of efflux pumps mediating the extracellular translocation of the antibiotic, to antibiotic inactivation or modification via the expression of modifying enzymes (see [117,118,119,120] for reviews on this topic). In recent years, daptomycin, a lipopeptide that interferes with the bacterial cell envelope function [121], has become a last-resort antibiotic used to treat infections brought about by MDR strains. While most strains are sensitive to daptomycin, non-susceptible strains are rising [122], and it has also been reported that biofilm-associated strains display increased tolerance to this antibiotic [122].

The overview provided above underlines the significant risk for public health posed by the rise of antibiotic resistance worldwide. It has been estimated that several millions of people could die prematurely in the next decades if antimicrobial resistance (AMR) is not tackled efficiently [123,124]. At the base of AMR, the excessive and/or inappropriate use of antibiotics in human and animal medicine has resulted in the selection for resistant strains. Coupled with poor hygiene conditions in healthcare settings and the food chain, this ultimately facilitates the spread of these strains within the population. In the last decades, the alarming rise of AMR, particularly in the clinical setting, prompted the development of surveillance strategies and global programmatic efforts [125]. Regarding *S. aureus* infections, antimicrobial stewardship has in fact led to a decreasing trend in nosocomial infections brought about by MRSA strains. Reports indicating such a descending trend were recently published from groups all across the globe, underscoring that AMR infections are a global threat, and consequently, antibiotic stewardship programs have been implemented worldwide [126,127,128,129]. However, an emerging threat, particularly in hospital-acquired infections, are coagulase-negative staphylococci (CoNS). CoNS cause hospital-acquired opportunistic infections, particularly linked to colonization of surgical wounds and prosthetic implants [130,131]. Among the most represented hospital-associated pathogenic CoNS, *S. epidermidis* and *S. haemolyticus* are responsible for the majority of bloodstream infections [132,133], and together with *S. capitis* and *S. hominis*, frequently cause infection outbreaks in neonatal care units worldwide [134,135,136,137]. CoNS infections are further complicated by the high percentage of methicillin and multi-drug resistance found in hospital-associated strains [138,139] and by their high propensity to form biofilms [140,141]. In this context, several studies have been shown that biofilm formation is a widespread characteristic of CoNS [142], and the expression of biofilm-promoting genes was found to be enriched in clinically-derived strains [143]. A contribution to the substantial morbidity caused by CoNS derives from the wide clonal distribution of such drug-resistant strains. Examples of this are the multi-drug clonal strains of *S. epidermidis* belonging to the genetic cluster 5 and *S. capitis* clone NRCS-A, which are globally distributed in clinical environments [134,144,145].

### 3.2. Limited Antibiotic Penetration in Biofilms

The extracellular matrix of biofilms can act as a shield, effectively hindering antibiotic penetration into biofilms. This makes it difficult for many antibiotics to reach cells present in the deepest layers, thus leading to a heterogeneous antibiotic exposure across the biofilm. Cells in the deepest layers of the biofilm may then be exposed to subinhibitory concentrations of antibiotics until they encounter a lethal dose. This gradual exposure activates the transcriptional response to low doses, which potentially promotes AMR mechanisms. Examples of antibiotics that have limited penetration into staphylococcal biofilms are vancomycin, oxacillin, cefotaxime and delafloxacin, while others such as amikacin and ciprofloxacin are not affected [146]. Penetration of antibiotics can be improved when biofilms are exposed to compounds that damage or disrupt the biofilm matrix, as it has been reported for delafloxacin [147].

In addition, the ECM also acts as reservoir for the accumulation of enzymes with the ability to inactivate antibiotics. In staphylococci, such enzymes include beta-lactamase [148] and aminoglycoside-modifying enzymes [149,150], among others. Notably, these proteins are enriched in the outer layers of the biofilm, offering frontline protection against antibiotics [151].

### 3.3. Heterogeneous Populations in Biofilms

Cells living in biofilms encounter different environmental conditions depending on their position in the biofilm. These conditions affect the global pattern of gene expression, resulting in a genetically identical population of cells with large physiological heterogeneity [152]. The spectrum of biofilm cells with different physiology is an effect of the diverse microenvironments that are produced within the biofilm; for instance, through the generation of chemical gradients [152]. One of the best-studied examples of a biofilm-associated chemical gradient is oxygen availability [153,154], which leads to dramatic effects on the expression pattern of gene clusters involved in, for example, metabolic pathways and controlled cell death (*cid*-*lrg* system) [153]. As a result, staphylococcal biofilms are characterized by containing cells with different metabolic profiles: cells growing with active respiration, cells growing fermentatively, dead cells and cells growing with a reduced metabolic activity (dormant cells) [154]. Such a heterogeneous pattern of growth within a biofilm contributes to the alteration of the susceptibility profile to antibiotics. Indeed, this heterogeneity promotes the emergence of cell subpopulations with physiological characteristics which render them resilient to certain antibiotics [153].

Related to this aspect, it is important to note that many antibiotics target only actively growing cells and require an active metabolism of the target cells to be functional. Therefore, dormant cells, characterized by a low metabolic activity and a slow growth phenotype, are generally resistant to many different antibiotics.

Tolerant and dormant cells, the latter also referred to as persister cells, can withstand high antibiotic concentrations, and are commonly found in staphylococcal biofilms. Tolerant cells are able to survive at high antibiotic concentrations during a transitory period without affecting the minimal inhibitory concentration [155,156]; this ability is a consequence of a mutation or environmental conditions. In contrast, persisters are a small bacterial subpopulation with the ability to survive adverse conditions. This characteristic is therefore not linked to genetic mutations, but instead to a temporary phenotypic variation. *S. aureus* persister cells develop stochastically when ATP levels are decreased during the stationary phase [45,157]. Indeed, biofilms are often characterized by a low ATP content, and cells residing in biofilms have been shown to have properties of persister cells [158,159]. How such persistent and tolerant cells are regulated at the molecular level is not completely understood; however, several metabolic pathways are likely to play an important role [160,161].

### 3.4. Biofilms as a Mediator of Horizontal Gene Transfer

It is also worth considering staphylococcal biofilms as potential sources for the spread of antibiotic-resistance genes to susceptible cells by horizontal gene transfer (HGT) [162]. In this process, DNA can be transferred horizontally between cells by phage transduction, conjugation or natural transformation. The latter mechanism, which involves the uptake of naked DNA from the environment, is probably very inefficient in *S. aureus* [163], whereas phage transduction and conjugation are therefore the most prominent mechanisms of HGT in staphylococci [164]. The high concentration of cells within a biofilm may also have a role in promoting HGT, particularly through conjugation-mediated genetic transfer, which occurs when cells are in close proximity [162]. As mentioned above, biofilms frequently have a polymicrobial nature; consequently, genes encoding resistance determinants can also be transferred between different bacterial species. Resistance to vancomycin in *S. aureus*, for instance, emerged as a consequence of the acquisition of the *vanA* gene from *Enterococcus* species [110,165]. Further research is needed to understand the importance of the biofilm-associated microenvironment as a mediator of horizontal gene transfer in staphylococci.

## 4. Molecular Targets to Fight Staphylococcal Biofilms

As discussed above, biofilm infections are commonly treated by using antimicrobial agents that target the biofilm-associated cells. In these cases, essential cellular processes and structures such as DNA replication, protein translation, and cell-wall and membrane integrity are targets for antimicrobial molecules [166,167]. Targeting such essential processes will eventually lead to cell death; however, such treatments may also result in the development of and selection for antibiotic-resistant or antibiotic-tolerant subpopulations. Particularly, since antimicrobial treatment of biofilms often entails prolonged antibiotic exposure, the probability to develop antibiotic resistance is also increased. Care should therefore be taken when treating biofilms using antimicrobials with high potential for resistance development. This can be accomplished by choosing agents characterized by modes of action that reduce the likelihood of resistance development (i.e., lipoglycopeptides), or adopting a combination therapy (e.g., multiple antimicrobial agents with different modes of action) to treat the infections (see Section 5 for examples of anti-staphylococcal agents that show high antimicrobial activity in biofilms).

An alternative to the conventional antimicrobial strategy is the inhibition of biofilm formation by means of anti-biofilm strategies [168]. Anti-biofilm strategies do not inhibit bacterial growth and division, but instead target molecules and pathways involved in the formation and maturation of biofilms without necessarily killing biofilm-associated cells. As a consequence, the selection pressure for antibiotic-resistance development will be lowered. Also, since factors involved in staphylococcal biofilm formation are highly species-specific (compared to targets for conventional antibiotics), anti-biofilm strategies may allow for development of narrow-spectrum precision agents, which will have low or no influence on other microbiota [168]. All the different stages of biofilm formation (see Figure 1 and Section 2) are potential targets for anti-biofilm agents. By targeting the attachment and/or multiplication stage, the formation or maturation of the biofilm will be inhibited. For example, altering the molecular composition or charge of a surface may hinder the attachment stage, and thus stop further biofilm formation [23,24,25,26,27]. In this context, the development of therapeutic inhibitors of sortase A, a membrane protein involved in anchoring of surface-exposed proteins, have received great research attention, as the inhibition of this protein will greatly alter the surface properties of the bacterial cells [169,170,171,172]. Moreover, inhibition of the EPS has also become an attractive strategy to target the later stages of the biofilm-development process. By using agents that inhibit enzymes involved in EPS production (i.e., Ica and autolysins) [173,174,175], the biofilm matrix will not be properly structured and matured [168,173,174,175,176]. Targeting metabolic pathways also may be an effective approach, since the metabolism (respiration or fermentation) has been shown to be tightly linked to EPS production and biofilm structuring in *S. aureus* [177]. Finally, mature biofilms can also be disrupted by directly targeting the already formed EPS molecules. By degrading PIA, eDNA or proteins, the biofilms will disintegrate [178,179], and the cells will be released. Modulating quorum-sensing mechanisms such as the *agr* system is another way to promote dispersal of staphylococcal biofilms [79,180]. When the bacterial cells are no longer associated with biofilms, they can potentially be eradicated by the host’s immune system. Furthermore, since dispersed bacterial cells are also more susceptible to antibiotics than biofilm-associated cells, a combined treatment of anti-biofilm and antibiotics is likely to be more effective than a single treatment. Such a combination treatment is also important to prevent the risk of reinfection with cells that are dispersed from a biofilm unless they are killed.

## 5. Alternative Treatments of Staphylococcal Biofilms

In the following section, different alternative strategies for treatment of staphylococcal biofilms will be discussed.

### 5.1. Bacteriocins

**Figure 2:**
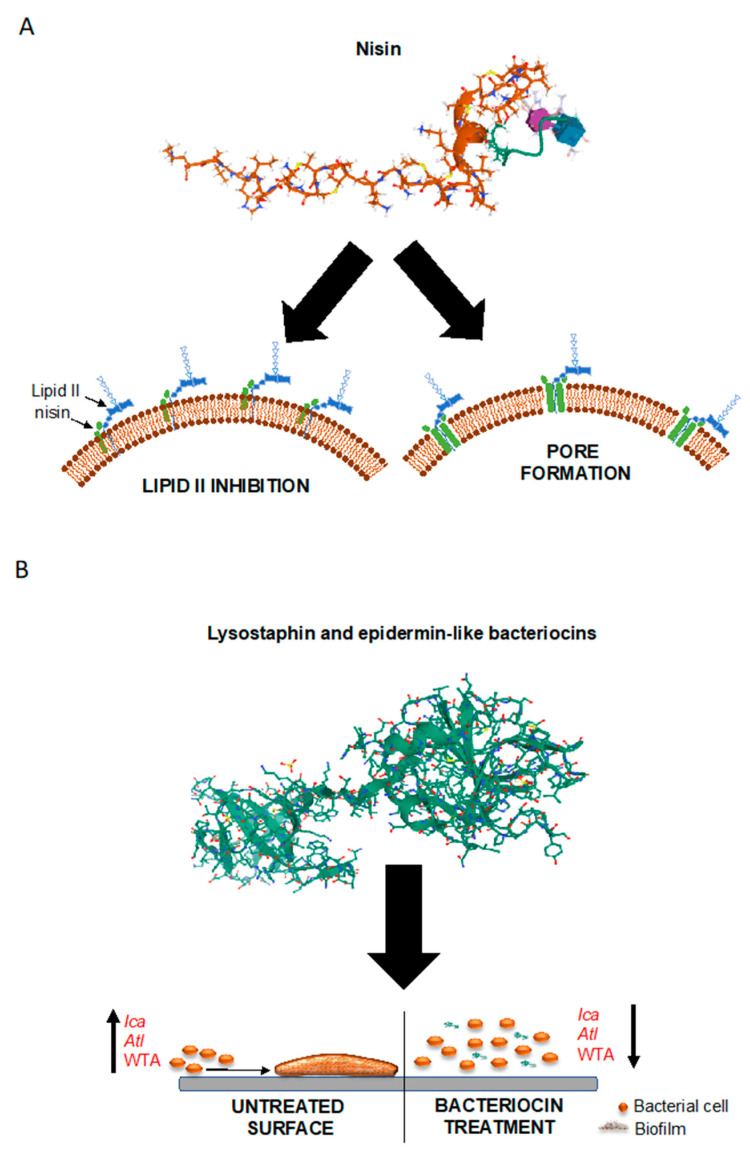
Model of anti-biofilm and bactericidal action of bacteriocins. (**A**) The structure of nisin in conjugation with lipid II is shown along with a representation of its dual mechanism of action. By interacting with lipid II molecules exposed on the cell surface, the bacteriocin inhibits the cell-wall biosynthesis and leads to pore formation in the bacterial cell membrane. (**B**) The structure of lysostaphin is shown as a representative example of staphylococcins. Its anti-biofilm action relies in part on the perturbation of wall teichoic acid (WTA), the function of which has been shown to be important in the process of biofilm formation. Epidermin-like bacteriocins (e.g., epidermin and gallidermin) follow a similar mode of action, and also lead to the downregulation of biofilm-promoting genes such as *ica* and *atl*. The protein structures were obtained from the Research Collaboratory for Structural Bioinformatics (RCSB) Protein Data Bank (PDP) (http://www.rcsb.org).

Bacteriocins are a class of naturally occurring peptides with antimicrobial properties. They are ribosomally synthesized by virtually all existent bacterial species in order to confer a competitive advantage for niche colonization [181,182,183]. Consequently, it is not surprising that many of these antimicrobials have activity against bacterial species closely related to the producer, although broad-spectrum bacteriocins do exist [182,184]. Bacteriocins are broadly subdivided into three classes, depending on whether they are modified at the post-translational level (class I) or not (class II), in addition to class III bacteriocins that are a group of large (M_r_ > 25 kDa) thermo-labile proteins [185,186,187]. Bacteriocins have gained increasing interest for their antimicrobial potency (being active in the nanomolar concentration range) and for their amenability to manipulation and bioengineering for increased target specificity, potency and improved physicochemical properties [188,189,190,191,192,193,194,195,196]. Most bacteriocins cause disruption of the membrane integrity on target cells, leading to leakage of the cell contents and membrane potential dissipation, and ultimately cell death. Thus, their mode of action differs from most antibiotics, which normally act as enzyme inhibitors. Due to these properties, bacteriocins have found their way into diverse practical applications, ranging from food preservation to medical applications; the latter including the treatment of antibiotic-resistant and biofilm-associated staphylococcal infections [197,198]. In this context, one group of bacteriocins, the lantibiotics, have received the greatest attention; among these, nisin is the most well-characterized member [196,199]. Nisin, together with other lantibiotics, is a potent inhibitor of cell-wall biosynthesis [200,201,202]; however, this peptide also induces pore formation in the target cell membrane; a dual mode of action that drastically reduces the frequency of resistance development (Figure 2A) [196,203]. It is interesting to note that the target molecule of nisin, as well as of some unrelated bacteriocins, is the cell wall precursor lipid II, the same as vancomycin. Importantly, however, since the binding sites for nisin and vancomycin differ, lipid II-binding bacteriocins retain their antimicrobial effects against vancomycin-resistant pathogens (Figure 2A) [204]. Studies on *S. epidermidis* and *S. aureus* biofilms have shown that nisin has a potent antimicrobial activity, but a limited ability to remove the biofilm itself [204,205,206]. However, bioengineering by targeted amino acid modifications has proved to be an effective strategy to improve its antibiofilm potential [195]. Bacteriocins produced by species belonging to the genus *Staphylococcus*, collectively known as staphylococcins, have also been described [207,208]. Most staphylococcins are class I bacteriocins (lantibiotics); among these, PEP5 and epidermin have been shown to inhibit the adhesion of *S. epidermidis* cells to silicone catheters in vitro [209], the initial step in biofilm formation and medical device colonization. A class III staphylococcin, the murein hydrolase lysostaphin [210] has been shown to have a potent anti-biofilm activity against *S. aureus* and *S. epidermidis* [211,212]. Interestingly, it has been successfully used to prevent biofilm formation on orthopedic implants in experimental animal models (Figure 2B) [213,214]. Gallidermin, a natural single amino acid variant of epidermin produced by *S. gallinarum* [215], also has been shown to possess a potent growth-inhibitory effect against *S. epidermidis* and *S. aureus* strains, and to prevent their biofilm formation [216,217]. It was suggested that the activity of gallidermin was linked to the inhibition of the biofilm-related genes *ica* and *atl* (Figure 2B).

**Figure 3:**
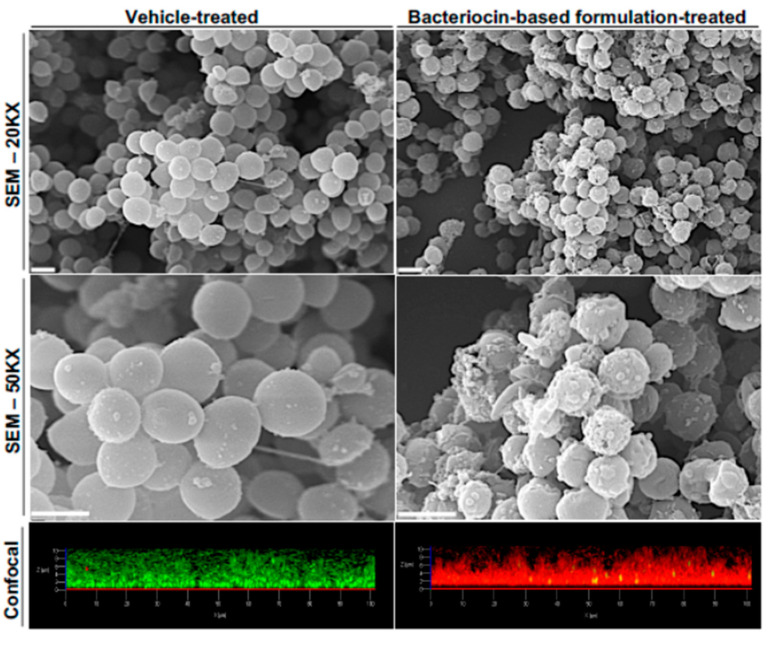
Visualization of the effects of a bacteriocin-based formulation on MRSA biofilms.

An important aspect linked to the use of bacteriocins as antimicrobial agents is their synergistic effect with antibiotics or other classes of bactericidal drugs. The advantages of this combinatorial strategy are several. Synergistic interactions between antimicrobials have been shown to reduce the concentrations needed to reach growth-inhibitory or biocidal effects against the target microorganisms, with consequent reduction of the risk of toxic effects, and to increase the spectrum of activity of the antimicrobials. In addition, as mentioned above, combinatory treatments also reduce the frequency of mutations conferring antimicrobial resistance to the treated strains. Several studies have highlighted the synergy between nisin and different classes of antibiotics [218,219,220,221] or other bacteriocins [222,223] against staphylococcal biofilms. In addition, synergistic effects between nisin or lysostaphin and bacteriophages have also been observed [224,225,226]. A recently discovered class-II bacteriocin, garvicin KS [227], also has been shown to be a potent inhibitor of *S. aureus in vivo*, when combined with penicillin G and the thiopeptide bacteriocin micrococcin P1 [228]. This antimicrobial combination has been shown to have a dramatic impact on the viability of the MRSA strain 33591 when used in a skin wound mouse model [197,228]. In addition, a similar garvicin KS-based formulation has been shown to be highly effective in eradicating established *S. aureus* biofilms in vitro [198]. In this regard, Figure 3 illustrates the effects of such bacteriocin-based formulation on MRSA biofilms produced in vitro, underlining its efficacy to disrup the viability of *S. aureus* biofilm-associated cells.

### 5.2. Phage-Derived Antibiofilm Strategies

**Figure 4:**
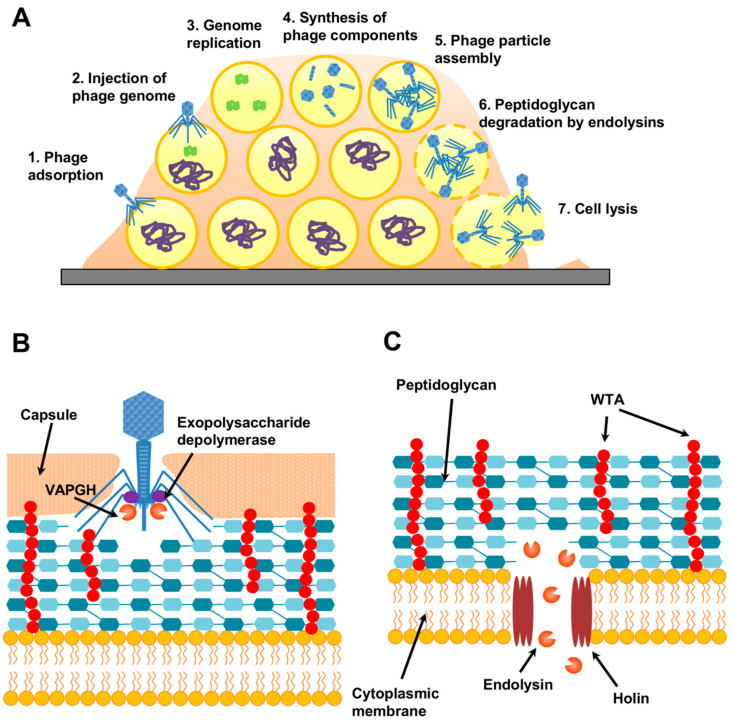
Bacteriophage replication through the lytic cycle. (**A**) Phage-infected *S. aureus* biofilm in which different cells are undergoing consecutive stages of the lytic cycle, while others remain uninfected. (**B**) Virion-associated proteins with antibiofilm potential that participate in early stages of the lytic cycle (VAPGHs and exopolysaccharide depolymerases). (**C**) Role of endolysins in cell lysis at the end of the lytic cycle. WTA, wall teichoic acids.

Treatment with bacteriophages (or phages), viruses that infect bacteria, is another promising strategy to fight *S. aureus* biofilms. Their antimicrobial activity stems from the fact that they generally kill their bacterial host at the end of the lytic cycle (Figure 4A).

The effects of the treatment with the bacteriocin formulation or its control vehicle [197] were visualized by scanning electron microscopy (SEM) (greyscale pictures—scale bar = 1 μm) and by confocal microscopy (color pictures—scale bar = 10 μm) using differential dye incorporation in order to mark living (green) and dead (red) cells. Note that the treatment with the formulation led to severe damage of the bacterial cells, with an apparent leakage of their content, more evident at high magnifications (50KX). Consistently, the treatment also led to a uniform switch in the pattern of dye incorporation, and therefore viability, of biofilm-associated cells for green (living) to red (dead). See reference [198] for methodological details.

Phages have recently gained attention because of their safe use in humans and for the environment, along with their high host specificity, thus not affecting the healthy microbiota. All known staphylococcal phages to date belong to the order Caudovirales, which consists of double-stranded DNA (dsDNA) tailed phages [229]. Depending on their tail structure, they can then be divided into three families, namely Siphoviridae (long, non-contractile tails), Myoviridae (long, contractile tails) and Podoviridae (short, non-contractile tails). Recently, the ICTV has included two new families: Ackermanviridae (contractile tails, related to the *Salmonella* ViI phage) and Herelleviridae (contractile tails, related to phage SPO1, Twort and K phages, phage P100 and ϕEF24C phage).

In order to be a potential antimicrobial candidate, a given phage must comply with certain prerequisites [230]. The most important condition is that it must be a virulent phage, as temperate phages are known to contribute to horizontal transfer of virulence and antibiotic-resistance markers. Another desirable property is that the phage exhibits a broad host range within its target species. Beyond that, in order to be a useful antifouling agent, the phage particles need to be able to reach and infect their host cells within the complex biofilm structure. In that sense, González et al. [231] demonstrated that two staphylococcal myophages, phiIPLA-RODI and phiIPLA-C1C, can move across the biofilm and propagate in susceptible sessile cells. Previous studies have already demonstrated the efficacy of both phages to kill biofilm-associated cells in mono- and mixed-species biofilms [232,233]. Other examples of phages that reduce biofilms formed by *S. aureus* include podoviruses CSA13 and SAP-2, as well as myoviruses phage K, DRA88 and LM12, among others [234,235,236,237,238]. Nonetheless, before applying phage therapy for antibiofilm purposes, the appropriate phage concentration for treatment must be determined. This is especially important, considering the evidence showing that low phage doses might promote biofilm development in *S. aureus* [239,240].

Notably, some staphylococcal phages possess polysaccharide-degrading enzymes (exopolysaccharide depolymerases) that can aid in removing the extracellular matrix of biofilms and bacterial capsules (Figure 4B) [233]. Such phages would, therefore, be particularly useful as part of an antibiofilm cocktail. Moreover, depolymerases themselves can be purified and used to complement different biofilm-elimination strategies, such as other phage-derived antimicrobials or antibiotics. For example, Olsen et al. [241] observed a synergistic activity between the poly-N-acetylglucosamine depolymerase DA7 and endolysin LysK for the removal of *S. aureus* biofilms.

Lytic proteins from phages can also be used as powerful antibacterial tools. Like phages, they are quite specific, being generally innocuous for non-target bacteria. On top of that, antistaphylococcal lysins do not easily select for resistant variants, as they can kill persister cells, which are fairly common in biofilms [242]. Phages have two types of lytic proteins: virion-associated peptidoglycan hydrolases (VAPGH) and endolysins, both of which can degrade peptidoglycan. However, while the first are used by the phage to partially degrade the cell wall and allow entry of the viral genome into the cell (Figure 4B), the latter are produced at the end of the lytic cycle in order to facilitate host-cell lysis and release of the newly formed viral particles (Figure 4C). *S. aureus* lysins have an additional perk—they possess a modular structure that enables easy design of truncated and chimeric proteins, which sometimes exhibit enhanced activity [243,244].

There are multiple examples of lytic proteins that can successfully kill biofilm-associated cells, including endolysins SAL200, SAL-2, phi11, PlySs2 (CF-301), PlyGRCS, LysH5 and LysRODI, as well as several engineered proteins like CHAPK, ClyH, CHAP-SH3b and ClyF [235,239,242,245,246,247,248,249,250,251,252]. Interestingly, besides their bactericidal activity, some staphylococcal phage lysins like CHAP-SH3b and LysH5 inhibit biofilm formation when present at subinhibitory concentrations [239,242]. This might be due to their downregulating effect on the expression of bacterial autolysins, some of which (AtlA and Sle1) are known to participate in biofilm development (see Section 2).

Bacteriophage-based antimicrobials are not only potential alternatives to antibiotics and disinfectants, but, like bacteriocins, are also good candidates for use in combination therapy. Indeed, many studies have shown the existence of synergy between phages and antibiotics for the treatment of biofilms formed by *S. aureus* alone or in multi-species biofilms [253,254,255,256]. In the case of disinfectants, however, the situation is slightly more complicated, as some phages can be inactivated by certain compounds, an important aspect to consider in the development of new antifouling products [257]. Phage lysins have also shown synergistic activity with antibiotics for the removal of biofilms formed by different *S. aureus* isolates, including some MRSA strains [258].

Although there is clearly a need for more studies to demonstrate the efficacy of phages and their derived proteins to eliminate staphylococcal biofilms in real-life scenarios, they certainly represent a viable, environmentally friendly option to reduce our overreliance on conventional antibiotics.

### 5.3. Antibodies

Staphylococcal infections trigger a natural response of the immune system by generating antibodies against a wide range of antigens, including surface proteins, toxins and cell-wall proteins [259,260,261,262]. Antibodies are highly specific, which brings the benefit of avoiding the damage to the host that may occur with other treatments. This characteristic reflects the potential of immunotherapy by therapeutic antibodies as a viable alternative narrow-spectrum antibiofilm treatment. Staphylococci have the particularity that not all strains express the same antigens; therefore, finding a universal target has been one of the challenges of immunotherapy, hence making vaccine development quite challenging [263]. Another potential disadvantage of antibodies, as with other antimicrobial agents, is that they might not be capable of penetrating into the deepest layers of a biofilm, and consequently, biofilms will not be disrupted completely. Nevertheless, a number of studies have shown the potential of disrupting staphylococcal biofilms by using antibodies targeting different staphylococcal antigens, such as surface proteins [264,265,266,267,268], cell-wall enzymes [269], PNAG [268] and toxins [266].

### 5.4. Nanotechnology

In recent years, the use of nanotechnology has also emerged as a promising alternative strategy to treat biofilms. Nanotechnology is based on the use of molecules in the range of 1–1000 nm [270], the small size of which allows them to penetrate the biofilm layers. Nanotechnology research on biofilm treatments has focused on two main areas: the use of nanoparticles with antimicrobial activity and the development of drug delivery systems. The first category includes the use of inorganic particles such as silver, zinc, titanium, copper and gold [271,272,273]. The nanotechnology-based drug-delivery systems have been shown to enable the slow and controlled release of drugs. Many systems have been generated and are based on different compounds such as lipids and polymers [7,274].

The use of nanotechnology offers multiple advantages in comparison to traditional treatments. For instance, materials with greater surface-area-to-volume ratios have improved reactivity. Furthermore, nanotechnology avoids problems such as enzymatic degradation, toxicity and unspecific delivery [273]. Although there are diverse benefits deriving from the use of nanoparticles, there is still a lack of studies regarding their interaction and toxicity in the human body, and long-term effects such as accumulation in tissues and organs [275]. Furthermore, as with traditional antibiotics, development of resistance is possible.

### 5.5. Photoinactivation

Photodynamic inactivation (PDI), also known as photodynamic therapy, is based on the use of visible light, a photosensitizer and oxygen to produce a phototoxic reaction that kills bacteria. Photosensitizers are light-sensitive dyes that can be activated by a proper wavelength; free radicals and reactive oxygen species (ROS) are produced after the photosensitizer reaches an excited state in the presence of oxygen. The free radicals and ROS produce oxidative damage in the bacteria. Molecules such as DNA, RNA, proteins and lipids are common sites for oxidative damage, and as a result, many essential pathways are affected. As many molecules are affected, it has been suggested that PDI treatments will not result in development of antimicrobial resistance.

PDI has been shown to be effective against staphylococci, including MRSA strains [276,277,278]. These studies showed the broad variety of photosensitizers with a destroying effect on biofilm development in vitro, making the use of PDI a promising treatment.

A variation of PDI is the use of antimicrobial blue light (ABL). Unlike PDI, ABL does not require the addition of a photosensitizer [279], as it takes advantage of the endogenous photoactivable metal-free porphyrins that usually accumulate within bacterial cells. ABL uses wavelengths of 400 to 470 nm to excite the porphyrins and produces a phototoxic reaction. ABL also has been shown to be effective against several bacterial strains and polymicrobial biofilms, including *S. aureus* [279,280], where ABL was used in vitro to effectively reduce bacterial viability.

### 5.6. Other Anti-Biofilm Agents

As mentioned above, cell-cell communication by quorum-sensing (QS) is important for staphylococcal biofilm formation, and such a system is an attractive target for anti-biofilm agents. Molecules that inhibit activation of the *agr* system have been identified, including natural products [281] and derivatives of the AgrA/AIP peptides, which compete for binding to AgrC and thereby block the activation of the regulatory pathway. It should be noted, however, that such quorum-quenching approaches may have unpredictable effects. For instance, the *agr* operon is the master QS regulator implicated in the expression of virulence factors responsible for host matrix attachment, immune evasion and expression of toxins [85,282]; the impairment of the *agr*-mediated QS in *S. aureus* and *S. epidermidis* has been shown to enhance their ability to form biofilms (likely due to defects in the detachment phase of biofilm biogenesis) [77], but also to reduce the pathogenicity of staphylococcal infections in vivo [283,284,285,286,287,288]. Recently, quorum-quenching molecules have also been successfully used to inhibit *S. epidermidis* biofilm formation by preventing the adhesion of the bacterial cells onto surfaces [289]. Quorum-sensing also mediates the communication between different microbial species within a biofilm, making QS interference an attractive therapeutic strategy in this case as well. For instance, a recent screening for quorum-quenching molecules led to the identification of small molecules able to inhibit the biofilm formation of *C. albicans* and *S. apidermidis* in polymicrobial biofilms [289]. In addition to quorum-sensing modulation, small molecule inhibitors of other enzymes and molecules were also investigated for their potential as anti-biofilm agents [290]. This includes molecules inhibiting the enzyme sortase A [169,170,171,172] and the use of surfactants, such as quaternary ammonium salts, in order to coat surfaces and prevent bacterial adhesion and biofilm formation. The latter strategy has proven effective in inhibiting the adhesion of *S. epidermidis* on surfaces with different characteristics [291], potentially representing a strategy to prevent the bacterial colonization of medical devices. Similar chemicals have also been shown to efficiently remove mature *S. epidermidis* biofilms [291].

Enzymes that directly degrade EPS components have also been successfully employed against staphylococcal biofilms. Dispersin B has been shown to degrade the PIA polysaccharides, while DNases and proteinase K have been used to degrade eDNA and proteins, respectively, in biofilm EPS.

Finally, novel genetic approaches, such as CRISPR-Cas, could also have a potential future role in treatment of biofilm-associated infections [292]. CRISPR-Cas systems allow targeting and cutting of specific DNA sequences, and CRISPR-Cas systems delivered by modified bacteriophages have been shown to selectively eradicate virulent *S. aureus* strains from mixed populations, to inhibit staphylococcal biofilms in vitro and to be effective against *S. aureus* in soft-tissue infection and skin-colonization models [293,294]. Future research will unravel whether CRISPR/Cas or other genetic approaches can be further developed to find effective treatments of staphylococcal biofilm infections.

## 6. Conclusions and Future Perspectives

The treatment of staphylococcal biofilm infections is a huge challenge. As we have discussed in this review, biofilm-associated staphylococci have reduced susceptibility toward antibiotics as a result of a protective biofilm matrix and the phenotypic heterogeneity in the biofilm population, including non-growing and slow-growing cells. Along with the increased antibiotic resistance, this poses a major obstacle for successful biofilm treatment. This highlights the need to find alternative strategies and targets to fight staphylococcal biofilms. A number of alternative agents and methods have been presented in this review, and future research should aim at identifying others with different targets. Furthermore, it will be important to expand the repertoire of potential anti-biofilm targets in staphylococci. The development of novel genetic tools and resources, such as genome-wide transposon insertion libraries [295,296], will now allow for a high-throughput search for such targets among the non-essential genes. This, together with other newly developed technologies such as CRISPR interference (CRISPRi) knockdown approaches [297], will be important tools to elucidate the molecular mechanisms of biofilm-associated functions; for example, identifying genes associated with the release of eDNA [298].

Combinatorial therapies also show great potential, as highlighted in the work by Kranjec et al. [198]. Using combinations of antimicrobials with different modes of action is often the norm to gain highest synergy, both in terms of effective killing of target cells and of reducing the development of resistance. Another interesting finding is that some combinatory therapies re-sensitize pathogens to certain antibiotics to which they have become resistant when performing mono-therapies [197,198,299]. The molecular mechanism behind this is not yet well known, but this can be an invaluable approach to reuse the many antibiotics that have become useless due to antibiotic resistance.

## Data Availability

No new data were created or analyzed in this study. Data sharing is not applicable to this article.
